# Autosomal Dominant Retinitis Pigmentosa Due to Class B *Rhodopsin* Mutations: An Objective Outcome for Future Treatment Trials

**DOI:** 10.3390/ijms20215344

**Published:** 2019-10-27

**Authors:** Alexander Sumaroka, Artur V. Cideciyan, Jason Charng, Vivian Wu, Christian A. Powers, Bhavya S. Iyer, Brianna Lisi, Malgorzata Swider, Samuel G. Jacobson

**Affiliations:** Department of Ophthalmology, Scheie Eye Institute, Perelman School of Medicine, University of Pennsylvania, Philadelphia, PA 19104, USA; asumarok@pennmedicine.upenn.edu (A.S.); charng.j@gmail.com (J.C.); vivian.wu@pennmedicine.upenn.edu (V.W.); chpowers@sas.upenn.edu (C.A.P.); brianna.lisi@pennmedicine.upenn.edu (B.L.); mswider@pennmedicine.upenn.edu (M.S.)

**Keywords:** optical coherence tomography, photoreceptors, retinal degeneration, rods

## Abstract

Gene therapy for adRP due to *RHO* mutations was recently shown to prevent photoreceptor death in a canine model of Class B disease. Among translational steps to be taken, one is to determine a method to detect efficacy in a human clinical trial. The relatively slow progression of adRP becomes a difficulty for clinical trials requiring an answer to whether there is slowed progression of degeneration in response to therapy. We performed a single-center, retrospective observational study of cross-sectional and longitudinal data. The study was prompted by our identification of a pericentral disease distribution in Class B *RHO*-adRP. Ultrawide optical coherence tomography (OCT) scans were used. Inferior retinal pericentral defects was an early disease feature. Degeneration further inferior in the retina merged with the pericentral defect, which extended into superior retina. In about 70% of patients, there was an asymmetric island of structure with significantly greater superior than inferior ellipsoid zone (EZ) extent. Serial measures of photoreceptor structure by OCT indicated constriction in superior retinal extent within a two-year interval. We conclude that these results should allow early-phase trials of therapy in *RHO*-adRP to move forward by inclusion of patients with an asymmetric extent of photoreceptor structure and by monitoring therapeutic effects over two years in the superior retina, a reasonable target for subretinal injection.

## 1. Introduction

Understanding the group of retinal diseases named retinitis pigmentosa (RP) has increased remarkably since it was first recognized by symptoms and ophthalmoscopy more than one and a half centuries ago. By fundus description, RP was characterized as initially showing abnormal pigmentation in the post-equatorial region [1]. Histopathology of human post-mortem donor retinal tissue from RP patients [2,3] moved the field from macroscopic to microscopic levels and revealed the complex outer and inner retinal cell abnormalities underlying the diseases. Molecular identification of the many monogenic causes of RP [4,5,6] led not only to more specific diagnoses but also to the current era when gene-specific therapies are being considered, initiated, or even ongoing [7,8].

Major advances in understanding RP disease mechanisms have also resulted from studies of animals with inherited retinal degeneration [9,10]. From a limited number of “animal models” of RP with unknown genetic causes, there was subsequent identification of the molecular basis of many of these retinal degenerations. Genetically engineered retinopathies that simulate human forms of RP followed. Studies revealing mechanisms also came from noninvasive electrophysiological and psychophysical methods in RP patients [6].

With all of these gains in understanding RP, is there progress toward clinical trials of treatment? For RP patients at advanced disease stages secondary to photoreceptor cell loss, there is an electronic retinal prosthesis [11], and there are optogenetic approaches being developed [12]. Leber congenital amaurosis (LCA), an early-onset autosomal recessive retinal blindness, can be grouped within the family of RP diseases, and there has been recent progress in gene-specific therapies. Patients with *RPE65* and *CEP29*0 forms of LCA show dissociation of structure and function, meaning greater visual loss than can be accounted for by residual photoreceptors [13,14,15] and were thereby strong candidates for improvement of vision with gene therapies. The *RPE65*-LCA clinical trials were focal subretinal injections using adeno-associated viral vectors to deliver the normal gene [16,17,18,19]. The *CEP290*-LCA clinical trial used intravitreal injections of an antisense oligonucleotide to restore correct splicing [20]. Improved vision was reported in trials involving LCA patients with mutations in either gene.

The goal of therapy for most forms of RP would be to slow the progression of retinal degeneration. This was the goal of earlier nutrient trials in RP, and results (using retinal or visual function) were able to be measured in terms of several years [21,22,23]. The pursuit of a shorter timeline to decision about efficacy prompted the use of transition zone constriction in XL (X-linked) RP patients with *RPGR* mutations. The width of the transition zone was defined on central horizontal optical coherence tomography (OCT) scans as the disappearance of a specific layer associated with the junction between inner and outer segments (IS/OS) or alternatively with the ellipsoids of the inner segments (ellipsoid zone or EZ). The EZ width was shown to decrease within two years and provided hope for a shorter timeline to determine change using this objective measurement [24]. The current work expands upon this seminal finding using retinal structure but in ad (autosomal dominant) RP due to *RHO* (*Rhodopsin*) mutations falling in the category of Class B [25,26]. This work was prompted by recent evidence from a canine model of *RHO*-adRP indicating that a mutation-independent gene therapy can prevent photoreceptor loss [27].

## 2. Results and Discussion

### 2.1. Autofluorescence (AF) Imaging as an Outcome Measure in RHO-adRP

All patients in the present study had adRP with Class B *RHO* mutations (Supplementary Table S1). Our recent observations indicated multiple components to the phenotype of Class B *RHO*-adRP. By perimetry and OCT, there were patients with altitudinal (hemifield) losses, pericentral losses, diffuse rod sensitivity losses, two of these three expressions, or all three [26]. Altitudinal (also named sectoral) visual losses have long been associated with adRP [28], and when *RHO* mutations were identified as a molecular cause of the disease, there were many reports of this regional retinal distribution in *RHO*-adRP [25,29,30,31,32]. Pericentral retinal degeneration (PRD), considered a rare clinical entity unto itself, leads to an annular loss of function and structure from about 3° to 40° [33]. Among the molecular causes associated with PRD are *RHO* mutations [33,34,35].

**Figure 1:**
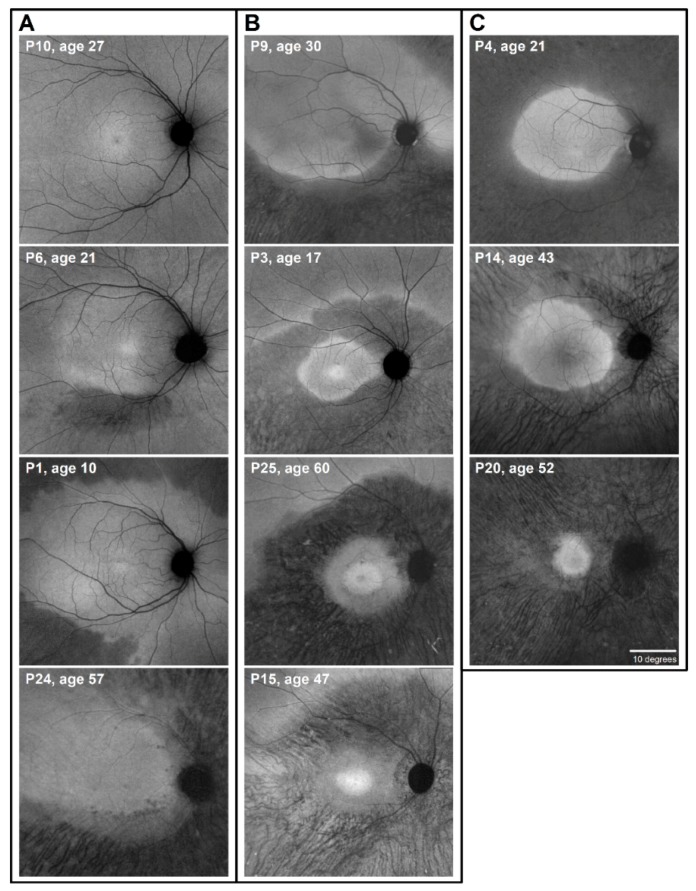
Topography of retinal pigment epithelial (RPE) disease in *RHO*-adRP. (**A**) Series of en face near-infrared (NIR) autofluoresence (AF) images (all displayed as right eyes for ready comparison) illustrating the spectrum from little or no RPE disease (P10); an abnormal pericentral inferior retinal region (P6); inferior and superior pericentral–midperipheral disease but sparing the nasal inferior retina (P1); and a more extensive inferior, nasal, and superior disease with superior temporal retention (P24). (**B**) Further images showing inferior retinal abnormalities and some superior nasal disease (P9); a pericentral ring of disease with inferior retinal extension at further eccentricities (P3); another pericentral ring with greater inferior abnormalities (P25); pericentral and greater inferior than superior retinal disease (P15). Different from the patients in (**A**) are the abnormalities in the central retina of patients in (**B**) and the considerable constriction of the central island in P15. (**C**) Patients with a larger central island surrounded by RPE disease (P4, P14). The retained central island of RPE is small in P20, and surrounding RPE is abnormal across the full extent of the image. Scale bar = 10 degrees.

Considering that short-wavelength AF (with 30° images) was recently reported to be an objective outcome in *RHO*-adRP patients [36,37], we examined the spatial topography of retinal pigment epithelial (RPE) abnormalities with AF imaging in our cohort of *RHO*-adRP patients. Although secondary to the primary photoreceptor disease, RPE abnormalities imaged by en face AF can provide a convenient view of the regional retinal variations in retinopathy (Figure 1). We performed near-infrared (NIR) AF [38] and examined 55° wide central retinal regions; NIR-AF was used to reduce any possibility of accelerating the natural history of *RHO*-adRP by excess absorption of light by rhodopsin [25,39,40]. There was a spectrum of RPE abnormalities: extracentral changes (usually more inferior than superior retina) (Figure 1A: P6, P1, P24; Figure 1B: P9), pericentral annuli that isolated a central island (Figure 1B: P3, P25, P15), and retained AF in central islands that were more constricted in some patients than in others (Figure 1C: P4, P14, P20). If AF would be useful as an outcome measure for a rod-based treatment, then attention would have to be paid to the extracentral changes we identified and not only the dimensions of the isolated central island [36,37]. The NIR-AF results confirmed the complexity of Class B *RHO* phenotypes [26] and further emphasized that a pericentral annulus can be a common pattern in this primary rod disease. The pericentral pattern, whether a complete annulus or incomplete, was present in 14 of the 31 patients (45%). Considering the normal distribution of rod photoreceptors and the location of the rod ring [41], it may not be surprising that rod cell loss manifests in this region.

### 2.2. Photoreceptor Layer Imaging with OCT: Cross-Sectional Observations

**Figure 2:**
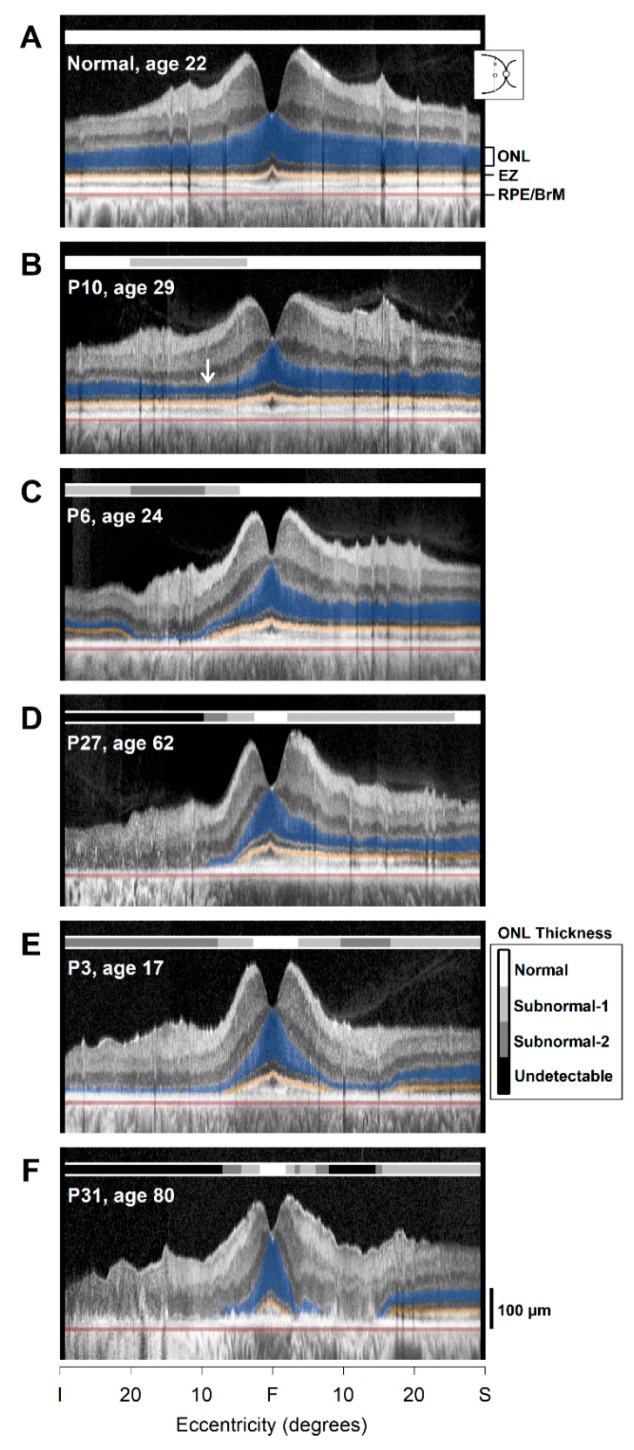
Disease sequence in *RHO*-adRP proposed from vertical optical coherence tomography (OCT) scans. (**A**) Normal scan through the fovea; photoreceptor outer nuclear layer (ONL), ellipsoid zone (EZ) line, and RPE/Bruch’s membrane (BrM) boundaries are labeled and colored for visibility: ONL (dark blue), EZ line (orange), RPE/BrM (dark red). Inset, location of the scan. Bars above scans (see key near E) indicate whether ONL is normal (white), subnormal (gray scale 1 = below the normal limit but <50%; gray scale 2 ≥ 50% below the normal limit), or undetectable (black). (**B**) Pericentral inferior ONL reduction (arrow) in P10. (**C**) Pericentral ONL loss inferior to fovea and less severe ONL reduction further inferior; fovea, pericentral superior, and further superior retina remain normal in P6. (**D**) Inferior ONL and EZ line are not detectable; superior pericentral ONL reduction is evident, but further superior retina is within normal limits in P27. (**E**) Central retinal integrity is retained, but there is markedly thinned ONL in the pericentral region and further inferior retina; pericentral superior ONL is also reduced, but there is greater thickness further superiorly in P3. (**F**) Small central island remaining with no detectable pericentral or inferior ONL; superior pericentral retinal ONL is also not detectable, but there is preserved ONL in the superior periphery in P31. I, inferior; S, superior; F, fovea.

Both pericentral and hemifield photoreceptor disease could be sampled with a vertical ultrawide (60°) OCT scan through the fovea. We studied this meridian in the *RHO*-adRP patients to try to predict a disease sequence (Figure 2). An OCT scan of a normally sighted subject showed thicker outer nuclear layer (ONL) at the foveal depression and thinner ONL across inferior and superior regions of the scan (Figure 2A). The EZ line extent was continuous across the entire scan length. P10 (age 29), with a G106R *RHO* mutation, showed a depression in ONL thickness at about 10°–20° inferior to the fovea but greater thickness further inferiorly; there was normal structure superiorly (Figure 2B). The EZ line appeared intact across the scan. P6 (age 24), with a T17M mutation, had a larger defect in the inferior ONL at 10°–20° and some loss of ONL thickness at further eccentricities inferiorly (Figure 2C). There was no detectable EZ line in the 10°–20° inferior region, but it was barely detectable at more inferior eccentricities. P27 (age 62), with a G106R mutation, had extensive loss of ONL and EZ line from 5° inferior to the fovea to the inferior retinal edge of the scan. Superior to the fovea at about 10°–25° eccentricity, there was a depression of ONL thickness (Figure 2D) but no discontinuity of the EZ line. This sequence of patient vertical scans suggested a pattern of progression of both pericentral and further inferior (presumed hemifield) retinal loss of photoreceptor layer integrity. Superior retinal preservation occurred within the vertical scans of P10 and P6, but there was an apparent beginning of pericentral loss in P27.

Further encircling of the fovea by degenerative change is illustrated in the OCTs of two other patients (Figure 2E,F). P3 (age 17), with a T58R mutation, had a substantial reduction of ONL thickness and loss of EZ line from about 10° inferiorly to the inferior edge of the scan. There was also ONL loss and a defect in EZ line at about 15° superiorly. ONL and EZ line were measurable again superiorly beginning at about 20° and continued to the limit of the scan length (Figure 2E). P31 (age 80), with a G106R mutation, had more extensive photoreceptor layer loss nearer to the fovea with only a small central ONL island remaining. ONL and EZ line became measurable at about 20° superiorly (Figure 2F).

To summarize, these observations in cross-sectional data from *RHO*-adRP patients using vertical ultrawide OCT scans suggest the following sequence of progression of photoreceptor degeneration: (1) inferior retinal changes appear in the pericentral region; (2) inferior retinal ONL and EZ losses occur (presumably later) at further eccentricities, likely representing the more recognized hemifield change; (3) superior retinal changes appear in the pericentral region; (4) asymmetrical constriction of the residual central island from the pericentral annulus toward the fovea (superior extent greater than inferior extent remaining); (5) relatively small circularly symmetrical central island remaining with or without peripheral retinal preservation.

### 2.3. From Observations to Quantitation of the Cross-Sectional OCT Data

**Figure 3:**
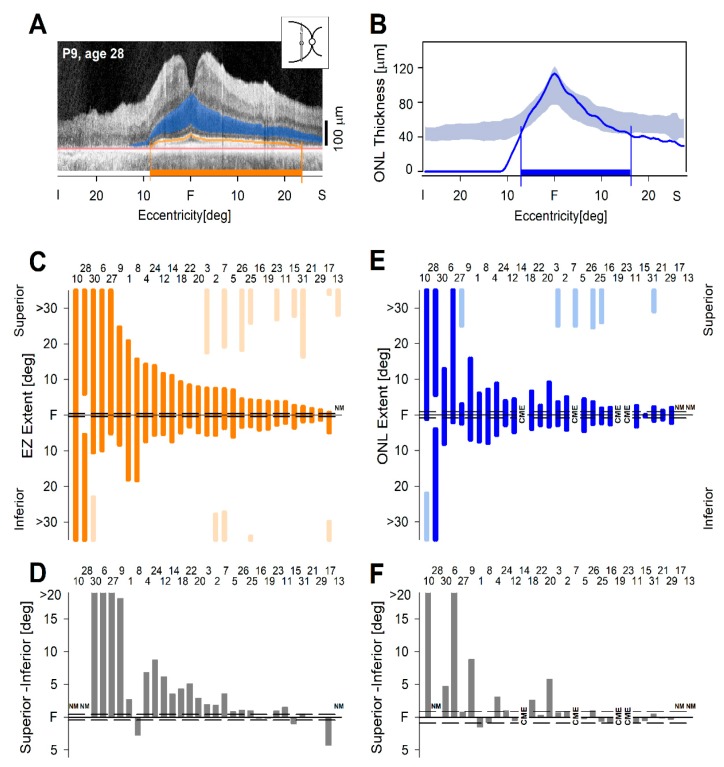
Asymmetry of photoreceptor parameters in vertical OCT scans of *RHO*-adRP patients. (**A**) Vertical scan across the fovea of a 28-year-old P23H patient; ONL, EZ line, and RPE inner boundary are labeled and colored for visibility: ONL (dark blue), EZ line (orange), RPE (light red). (**B**) Graph of ONL thickness (blue line) and ONL extent (blue bar on horizontal axis) in these regions; light blue band represents normal limits of ONL thickness. (**C**) Quantitation of EZ line extents from fovea into superior retina and fovea into inferior retina. Numbers above the bars in (**C**–**F**) correspond to patient numbers in Supplementary Table S1. Dark orange bars are the contiguous EZ regions from fovea superiorly and inferiorly. Note: Light orange segments are the eccentrically retained EZ regions at the edge of the scan. (**D**) The difference between EZ extents. (**E**) Quantitation of ONL thickness extents from fovea into superior retina and into inferior retina. Dark blue bars are the contiguous ONL regions from fovea superiorly and inferiorly. Note: Light blue segments are the eccentrically retained ONL regions at the edge of the scan. (**F**) Differences between the ONL extents. CME, cystoid macular edema precluded measurement.

Building on previous studies that measured ONL and EZ line in different forms of RP [24,26,42,43,44], we quantified these photoreceptor parameters in the vertical OCT scans of patients with *RHO*-adRP (Figure 3). As an example of the measurement, a vertical scan from a 28-year-old man with P23H *RHO*-adRP is illustrated (Figure 3A,B). ONL thickness and EZ lines are marked on the scan (Figure 3A); ONL limits are also graphed (Figure 3B).

EZ line measurements were made initially in one eye (chosen randomly, 14 right eyes, 17 left eyes) of the 31 patients (Figure 3C). There were two patients who had EZ extent for the full length of the scan. P13 had central retinal atrophy from chronic cystoid macular edema (CME) and only a small island of detectable EZ line superiorly. In the remaining 28 patients, there was measurable superior–inferior extent. Compared to intervisit variability [24], there was significantly greater superior than inferior EZ length extent in 20 (71%) eyes; three (11%) eyes had more inferior than superior extent, and five (18%) eyes either could not be measured or had no difference (Figure 3D).

The ONL extent was defined as the distance from the fovea until ONL became reduced below normal limits. All 31 patients were studied for superior and inferior extents as well as the difference between the two extents. The ONL results for the same eye as the EZ measures are plotted (Figure 3E). In four patients with severe CME, the ONL was unable to be quantified; P28 had normal ONL across the entire scan except in the central few degrees, where there were drusen. ONL extents were far less informative as to asymmetric length than the EZ line (Figure 3F).

Given a uniocular clinical trial in a rare disease, it is important to know if the contralateral eye can be used as an untreated control. Therefore, EZ line measurements were made in all contralateral eyes. The EZ extents are plotted for the right eye (RE) against those in the left eye (LE) (Supplementary Figure): superior extents of RE versus superior extents in LE and inferior extents of RE versus those of the LE. To determine if there was interocular agreement, the slope of linear regression and Pearson’s coefficient were computed. EZ extents in RE and LE showed a strong positive relationship, and the correlation was statistically significant (R = 0.947, *p* < 0.0001). Slope of the linear regression (dashed line is regression; dotted lines are 95% confidence intervals) was not different from slope of the equality line (solid line). Interocular symmetry was higher for the EZ extent up to 15° (95% of all EZ differences fell within ±2.40° interval) and lower at greater extents. For EZ extent in the range of 15°–30°, 95% of all EZ differences fell within ±8.44° interval.

### 2.4. Longitudinal OCT Data in RHO-adRP

Ideally, a single OCT scan in *RHO*-adRP would not only identify which patient should be included in a clinical trial but would also allow for quantitation of an outcome measure that changed from baseline in a two-year timespan and thereby determine whether a treatment was efficacious in halting progression. A subset of 22 of our cohort of 31 patients was examined on two visits separated by approximately two years (Supplementary Table S1). We postulated from the cross-sectional data that there would be asymmetrical constriction of the residual central island from the pericentral annulus toward the fovea, and loss of the superior extent could be more readily detectable.

**Figure 4:**
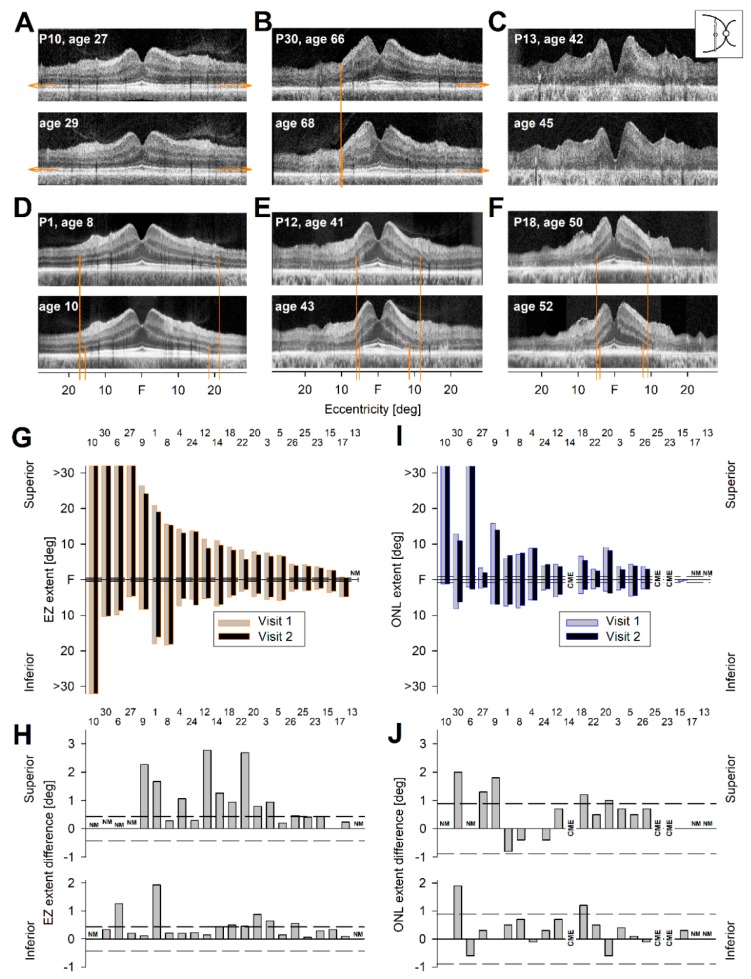
Serial vertical OCT scans over a two-year interval in *RHO*-adRP patients. (**A**–**C**) Serial scans illustrating the reasons why change in EZ extent could not be measured. (**A**) Vertical scan of a 27-year-old G106R patient at two timepoints separated by two years. Note: Left eye is used for this demonstration; right eye was used in Figure 2B. (**B**) Vertical scan in a 66-year-old P23H patient at baseline and two years later. (**C**) Vertical scan in a 42-year-old T58R patient at baseline and then two years later. (**D**–**F**) Serial scans showing measurable EZ extents in both superior and inferior retinal segments in 8-year-old, 41-year-old, and 50-year-old patients, all with P23H *RHO* mutations. (**G**) Quantitation of all 22 patients’ EZ extents from fovea into superior and inferior retina at baseline (gray with orange border) plotted adjacent to the subsequent visit two years later (black with orange border). Patient numbers are above the data in (**G**–**J)**. (**H**) Comparison of the differences between baseline and two-year interval data for EZ extent superiorly and inferiorly. Dashed lines indicate the intervisit variability limits. (**I**) Quantitation of all 22 patients’ ONL extents from fovea into superior and inferior retina at baseline (gray with blue border) plotted adjacent to the subsequent visit two years later (black with blue border). (**J**) Comparison of the differences between baseline and two-year interval data for ONL extent superiorly and inferiorly. Dashed lines indicate the intervisit variability limits. CME, cystoid macular edema; NM, not measurable (for different reasons; see text).

Beginning with EZ length, there were five patients with serial data who failed the ideal of a single informative scan that could be monitored for change in superior versus inferior extent (illustrated in Figure 4A–C). The reasons for failure were as follows: (1) EZ extent was present across the entire scan (Figure 4A; *n* = 1); (2) EZ extent was abnormally reduced inferiorly but was present across the entire superior length of the scan (Figure 4B; *n* = 3), thereby not allowing for a superior versus inferior extent comparison; and (3) there was no measurable EZ line at any location in the scan at the first visit (Figure 4C; *n* = 1). The remaining 17 patients had measurable superior and inferior EZ extents that were less than the full length of the scan (illustrated in Figure 4D–F). The data for EZ extent from fovea superiorly and inferiorly are graphed for both visits (Figure 4G). At the far left is a patient with full width of scan and then the patients with limited inferior retinal EZ extent but full extent superiorly (*n* = 3). At far right is a patient without measurable EZ line across the scan. Between these extremes are the 17 patients with measurable EZ extent in both directions, ordered from higher to lower by their superior retinal EZ extent at first visit. 

We then measured and plotted the differences in EZ extents between visits (Figure 4H). Of the patients with measurably different data superiorly, 10/17 (59%) showed differences that were outside the limits for intervisit variability, and there was no correlation with age (*r^2^* = 0.03). Of the patients with measurable difference data inferiorly, 6/20 (30%) showed differences that were outside the limits for intervisit variability. The asymmetric changes with superior length greater than inferior length mirrored the results from cross-sectional data analyses (Figure 3C). There was thus detectable and significant constriction of the EZ length from the superior retina extent to the fovea over the interval from baseline to two years. These differences between visits detected in the one eye were also present in the contralateral eye.

The ONL extents were also quantified to determine if this photoreceptor parameter could be used in place of or possibly to complement the EZ line data (Figure 4J). The results also showed asymmetries, but some of the data were complicated by CME and not as useful as those with EZ extent measures.

### 2.5. An Objective Outcome for a Clinical Trial of RHO-adRP

How can these observations be of practical use for a clinical trial attempting to slow the progression of rod photoreceptor degeneration in *RHO*-adRP? AF imaging in the current study proved useful to further illustrate the range of regional retinal disease expression, but there needs to be a method to quantify such results beyond measuring dimensions of a central island dominated by residual cone photoreceptors at late disease stages [36,37]. In a primary rod photoreceptor disease as *RHO*-adRP and especially in Class B patients with residual rods, ideally there should be a photoreceptor parameter that could measure rod photoreceptor integrity, like OCT. Based on results of the present study, we suggest that molecularly identified patients should be screened with vertical ultrawide OCT scans spanning 30° into the superior and 30° into the inferior retina, considering the wide availability of OCT and the good fixation of most of these patients. Patients with an asymmetric extent of ONL and EZ line, i.e., greater superior than inferior length, would be candidates for inclusion. Ideally, the vertical superior length should extend to at least 10° from the fovea. If an intended therapy was rod-specific, ONL thickness at 10° (or a further eccentricity leading into the transition zone of declining ONL) should be no less than about 50% of normal so as to include a substantial number of rod photoreceptors and not simply represent an extended cone central island. At 10° in the superior retina, for example, the normal density of rod photoreceptors is about 150,000/mm^2^, whereas cone density is only about 7500/mm^2^ [41].

Considering the many detailed studies of OCT photoreceptor parameters in recent years, what makes the present observations different? One of the most commonly used OCT analysis techniques for estimating disease progression is based on spatial movement of a disease–health transition boundary, also called the transition zone. Early observations using OCT about photoreceptor layer diminution in inherited retinal degenerations were in *RHO*-adRP and Usher syndromes [42,45,46]. The transition zone, as we defined it, is where the IS/OS peak (thought to originate near the junction between inner and outer segments or near the ellipsoids of the inner segments) becomes undetectable. The definition of the transition zone that we use is similar to the boundaries of EZ width [24], but our definition is based on the full dynamic range of the OCT backscatter signal as originally acquired. There are several OCT imaging studies evaluating the short-term progression of the EZ line relevant to Class B *RHO* patients. One study [43] measured EZ-width progression over approximately two years in a group of 33 adRP patients, 19 of which had known *RHO* mutations, and 13 of which had the common Class B P23H mutation. In this cohort, the total EZ width changed by 0.4° ± 0.5°/year and 0.3° ± 0.5°/year along the horizontal and vertical meridians, respectively; 95% confidence interval of test–retest repeatability was 0.9°.

Another study measured EZ-width progression over approximately two years in a group of 71 RP patients, 19 of which had adRP, and two of which had known *RHO* mutations [47]. Overall EZ-width progression averaged 0.45°/year (130 µm/year with SE of 11 μm); the 95% confidence interval of test–retest repeatability was 243.8 um (~0.85°). A third study measured EZ-width progression over ~4.5 years in a group of 10 adRP patients, all with *RHO* mutations but not necessarily all with Class B mutations [36]. Overall EZ-width progression averaged 0.5°/year (152 μm/year with SE of 37 μm); the 95% confidence interval of test–retest repeatability was 92.5 μm.

Importantly, previous studies have measured EZ width as the distance from one transition boundary to another transition boundary. This assumes that boundaries constrict symmetrically; a single transition boundary (in temporal, superior or inferior directions) would be expected to move at about half the rate of the EZ width measures provided: on average 0.15°–0.25°/year based on the three studies mentioned above. The current study measured separately the segments from fovea to superior and to inferior retina because of the observation of asymmetry in most of the vertical scans recorded from these patients. The transition along the inferior retina had the slowest progression rate at 0.26 (±0.26)°/year and the transition along the superior retina had the fastest progression rate at 0.48 (±0.42)°/year. Although we did not analyze the horizontal meridian, the temporal retina may be useful to include in further studies. Interpretation of nasal retinal scans, however, can be complicated because of variable crossing of the optic nerve depending on retinal anatomy and slight changes in head tilt. Our results from the inferior retina are near the upper estimates of the current literature, whereas our estimate in the superior retina is approximately twice as fast. A potential contributor for this apparent discrepancy is that the measurement of EZ width in the literature dilutes the movement of individual transition zones by combining the progression in two distinct regions of the retina. Another contributor could be our inclusion of only patients with known Class B *RHO* mutations; there is no direct correspondence in the literature for this. Of note, this strategy also leads to the suggestion of targeting subretinal treatment to a wide superior region (up to but not including the fovea and its usually well-preserved cone vision and visual acuity) with the goal of assuring long-lasting rod and secondarily cone function in the inferior field of vision.

Beyond the observations made in this study, there is a need to evaluate measures of visual function (subjective and objective) as well as other measures of retinal structure. The current manuscript did not attempt to compare and correlate several outcome measures—a worthwhile pursuit in its own right. Our scope was limited to the single outer retinal structural outcome that we feel is novel and may be very useful to demonstrate measurable disease progression in a two-year interval. Once the findings are confirmed and extended in large, possibly multicenter, natural history studies of this specific RP genotype and phenotype, there may be sufficient evidence to begin translation to a clinical trial of the proof-of-concept results in the canine model of *RHO*-adRP indicating that a mutation-independent gene therapy can prevent photoreceptor loss [27].

## 3. Summary and Conclusions

The *RHO* gene was the first genetic cause identified for RP, and over 100 mutations were subsequently discovered. Only recently, however, was a mutation-independent *RHO* gene therapy shown to prevent photoreceptor death in a large animal model of the disease. Despite this major step toward feasibility of a human clinical trial, *RHO*-adRP (specifically, Class B) remains a slowly progressive degeneration and, given the safety of a therapy, detecting efficacy in a reasonable time is a high hurdle. We made serial measures of photoreceptor structure in molecularly identified *RHO*-adRP patients and followed a subset for two years. These studies indicated that a specific OCT feature of the disease was changing within this interval. An objective outcome using common instrumentation should accelerate the path toward clinical trials of treatment.

## 4. Materials and Methods

### 4.1. Subjects

Patients with *RHO* gene mutations were included in this study (*n* = 31); a subset of these patients (*n* = 21) returned for a re-evaluation in about 2 years (range, 2.0–2.7 years). Molecular testing of the patients and their families has been previously reported [25,26] (see Supplementary Table S1). Procedures followed the Declaration of Helsinki, and the study was approved by the Institutional Review Board (IRB). Informed consent, assent, and parental permission were obtained, and the work was HIPAA-compliant.

### 4.2. Near-Infrared Autofluorescence Imaging

A confocal scanning laser ophthalmoscope (HRA2 or Spectralis HRA without OCT, Heidelberg Engineering, Heidelberg, Germany) was used to record near-infrared reduced-illuminance autofluorescence imaging (NIR-RAFI or NIR-AF) as previously described [38]. In brief, images were obtained with 790 nm NIR excitation light (100% ICG laser power setting; and 95% or 105% detector sensitivity for HRA2 or Spectralis HRA, respectively; automatic normalization off), and emission signals were collected between 810 and 900 nm. High speed mode was used for acquisition, and multiple images were registered and averaged to increase the signal-to-noise ratio. Images obtained with 30° and 55° lenses were used.

### 4.3. Optical Coherence Tomography

OCT was performed with a spectral–domain (SD) system (RTVue-100; Optovue Inc., Fremont, CA, USA). For analysis of ONL and EZ line extent, overlapping OCT scans (30 degrees in length; 1019 longitudinal reflectivity profiles (LRPs), each averaging 17–32) were used to cover the vertical meridian up to 30 degrees eccentricity from the fovea. Post-acquisition processing of the data was performed by one of the authors (A.S.) using custom programs (MATLAB 2018a, MathWorks, Natick, MA, USA). LRPs making up the scans were aligned by straightening the major RPE reflection. ONL layer thicknesses in patients were quantified [44,48], plotted as a function of eccentricity, and compared with the normal ranges (mean ± 2SD; *n* = 15; age range 8–62 years). EZ line extent was determined as previously published [44].

## Supplementary Materials

Supplementary materials can be found at https://www.mdpi.com/1422-0067/20/21/5344/s1.
