# Supplementary material for: Autosomal Dominant Retinitis Pigmentosa Due to Class B Rhodopsin Mutations: An Objective Outcome for Future Treatment Trials
Source: Int J Mol Sci. 2019 Oct 27;20(21):5344. doi: 10.3390/ijms20215344 (PMC6861901; doi:10.3390/ijms20215344)
Supplement: Supplementary file 1 [file ijms-20-05344-s001.pdf]

Supplementary Material

Autosomal Dominant Retinitis Pigmentosa due to Class B *Rhodopsin* Mutations: an Objective Outcome for Treatment Trials

Supplementary Table S1. Characteristics of the adRP-*RHO* Mutation Patients.

Patient No./ Gender	Protein Change	Age at Visit	Years Between Visits	Best Corrected Visual Acuity	
				RE	LE
P1/F	Pro23His	8	2.0	20/25	20/25
		10		20/25	20/25
P2/M	Gly89Asp	10	-	20/20	20/20
P3/M	Thr58Arg	17	2.7	20/20	20/20
		20		20/20	20/20
P4/F	Pro23His	21	2.0	20/20	20/20
		23		20/20	20/20
P5/M	Asp190Tyr	22	-	20/25	20/32
P6/M	Thr17Met	21	2.1	20/20	20/20
		24		20/20	20/20
P7/F	Thr17Met	24	-	20/32	20/32
P8/F	Pro23His	25	2.0	20/20	20/20
		27		20/20	20/20
P9/M	Pro23His	28	2.0	20/20	20/20
		30		20/20	20/20
P10/M	Gly106Arg	27	2.3	20/20	20/20
		29		20/20	20/20
P11/M	Gly89Asp	38	-	20/25	20/25
P12/F	Pro23His	41	2.0	20/20	20/20
		43		20/20	20/20
P13/M	Thr58Arg	42	2.7	20/125	20/400
		45		20/160	20/500
P14/F	Pro23His	43	2.0	20/32	20/25
		45		20/32	20/25
P15/F	Thr58Arg	47	2.0	20/32	20/125
		49		20/50	20/125
P16/F	Glu344Ter	48	-	20/20	20/25
P17/F	Thr58Arg	49	2.5	20/32	20/80

		52		20/50	20/100
P18/F	Pro23His	50	2.0	20/20	20/20
		52		20/20	20/20
P19/F	Glu344Ter	52		20/32	20/20
P20/M	Pro23His	52	2.0	20/20	20/20
		54		20/20	20/20
P21/M	Thr342Met	53		20/25	20/25
P22/F	Pro23His	55	2.0	20/20	20/25
		57 [a]		20/20	20/20
P23/F	Gly106Arg	56	2.3	20/20	20/32
		58		20/32	20/40
P24/M	Pro23His	57	2.0	20/20	20/20
		59		20/20	20/20
P25/F	Gly106Arg	60	2.1	20/32	20/20
		62		20/32	20/20
P26/M	Thr17Met	58	2.1	20/20	20/25
		60		20/20	20/32
P27/F	Gly106Arg	60	2.1	20/20	20/20
		62		20/20	20/20
P28/M	Thr193Met	64		20/20	20/63
P29/M	Pro23His	65	-	20/250	20/20
P30/F	Pro23His	66	2.0	20/20	20/25
		68		20/20	20/32
P31/M	Gly106Arg	80	-	20/40	20/20

[a] patient had cataract surgery, both eyes

Supplementary Material

Autosomal Dominant Retinitis Pigmentosa due to Class B *Rhodopsin* Mutations: an Objective Outcome for Treatment Trials

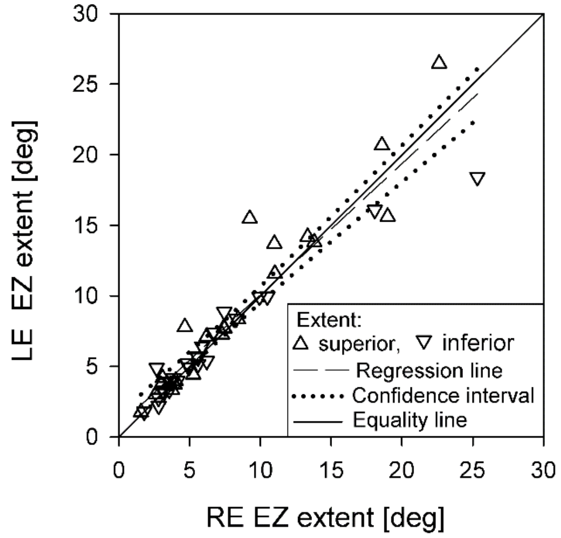

Supplementary Figure.

Interocular comparison of EZ extents. EZ extent for both superior and inferior direction in LE plotted against EZ extents in RE. Dashed line is linear regression, dotted lines are 95% confidence interval. Solid line is equality line.
